# Evaluation of the Idylla IDH1-2 Mutation Assay for the Detection of *IDH* Variants in Solid Tumors and Hematological Malignancies

**DOI:** 10.3390/ijms27021017

**Published:** 2026-01-20

**Authors:** Pauline Gilson, Marc Muller, Guillaume Gauchotte, Smahane Fadil, Marie Husson, Idrissia Hanriot, Andréa Witz, Julie Dardare, Margaux Betz, Jean-Louis Merlin, Alexandre Harlé

**Affiliations:** 1 Institut de Cancérologie de Lorraine, Centre National de la Recherche Scientifique (CNRS) Unité Mixte de Recherche 7039 (UMR 7039), Université de Lorraine, 6 Avenue de Bourgogne, CS 30519, 54519 Vandœuvre-lès-Nancy, France; m.husson@nancy.unicancer.fr (M.H.); m.betz@nancy.unicancer.fr (M.B.); jl.merlin@nancy.unicancer.fr (J.-L.M.); a.harle@nancy.unicancer.fr (A.H.); 2 Service de Biologie Moléculaire des Tumeurs, Département de Biopathologie, Institut de Cancérologie de Lorraine/CHRU Nancy, Rue du Morvan, 54511 Vandœuvre-lès-Nancy, France; marc.muller@chru-nancy.fr (M.M.); s.fadil@nancy.unicancer.fr (S.F.); i.hanriot@chru-nancy.fr (I.H.); a.witz@nancy.unicancer.fr (A.W.); j.dardare@nancy.unicancer.fr (J.D.); 3 Laboratoire de Génétique, Centre Hospitalier Universitaire Nancy, Rue du Morvan, 54511 Vandœuvre-lès-Nancy, France; 4 Service d’Anatomie et Cytologie Pathologiques, Tumorothèque BB-0033-00035, Département de Biopathologie, Institut de Cancérologie de Lorraine/CHRU Nancy, Rue du Morvan, 54511 Vandœuvre-lès-Nancy, France; g.gauchotte@chru-nancy.fr; 5 INSERM U1256 NGERE, Université de Lorraine, 54500 Vandœuvre-lès-Nancy, France

**Keywords:** isocitrate dehydrogenase, polymerase chain reaction, immunohistochemistry, next-generation sequencing

## Abstract

Isocitrate dehydrogenase (*IDH*) variants can lead to the development and/or progression of various solid tumors and hematological malignancies. *IDH* testing can guide diagnosis, prognosis, and therapeutic choice and typically relies on NGS, IHC, or PCR-based assays. Here, we evaluated the analytical performance of the Idylla IDH1-2 mutation assay for *IDH* variant detection using 70 fixed samples from patients with solid tumors and 36 DNA extracts from patients with acute myeloid leukemias previously characterized by NGS +/− IHC. Idylla IDH1-2 mutation assay gave 98.1% of valid results with an overall agreement, sensitivity, and specificity of 97.1%, 96.2%, and 98.1%, respectively, compared to NGS. Using commercial DNA standards, the limit of detection of the assay was 1.6% and 0.5% for *IDH1* R132H and *IDH2* R172K variants, respectively. Based on these data, the Idylla IDH1-2 mutation assay represents a fast and reliable alternative to detect *IDH* hotspot variants in solid tumors and hematological malignancies using either fixed tissue sections or DNA extracts. Particular attention, however, is needed for the interpretation of cases with cycle of quantification values of the internal controls over 35, for which a variant with low allelic frequency could be missed due to low DNA quantity or quality.

## 1. Introduction

Isocitrate dehydrogenase (IDH) enzymes are implicated in major cellular processes, including the Krebs cycle, lipid metabolism, epigenetic regulation, DNA repair, and redox homeostasis. Three different isoforms (IDH1, IDH2, and IDH3) are present in humans and catalyze the conversion of isocitrate to α-ketoglutarate (α-KG) by oxidative decarboxylation in different cellular compartments.

*IDH* variants are observed in various solid and hematological tumor types, including gliomas (Gs, >70%), chondrosarcomas (CSs, 50%), cholangiocarcinomas (CCs, 15–20%), solid papillary breast carcinomas with reverse polarity (SPBC, 77–80%), and acute myeloid leukemias (AMLs, 10–15%) [1,2,3]. Gain-of-function variants mostly arise in the catalytic domain of the enzymes, notably at arginine 132 in *IDH1*, and arginines 140 and 172 in *IDH2*. The frequency and repartition of *IDH* variants differ between tumor types [1]. Mutated IDH proteins acquire a neomorphic activity and favor the consumption of α-KG to produce and accumulate the oncometabolite D-2-hydroxyglutarate (D-2-HG) [1]. Due to its high structural homology with α-KG, D-2-HG can inhibit, by competition, α-KG-dependent enzymes, resulting in dysregulation of the epigenetic and metabolic processes, redox imbalance, and initiation of tumorigenesis.

*IDH* variants are associated with better prognosis in Gs and are considered key biomarkers to classify central nervous system tumors [4,5]. Conversely, the presence of *IDH* variants does not seem to be correlated with clinical outcomes in patients with CCs [6,7]. The prognostic value of *IDH* variants remains controversial in CSs [8,9,10,11,12]. In AML, the sole *IDH* status does not seem to have prognostic significance, although few studies reported more favorable outcomes with *IDH2* variants and poorer prognosis with *IDH1* variants [13,14,15,16,17,18,19,20]. In recent years, targeting *IDH* variants has been actively investigated given their high prevalence; their occurrence early in solid tumor development and the uniform expression of the coded mutated protein in tumor tissues [21]; and their crucial role for disease progression in hematological malignancies [22]. Several allosteric inhibitors of mutated IDH have been developed and evaluated during preclinical studies and clinical trials. To date, three IDH inhibitors have been sequentially approved by the US Food and Drug Administration (FDA): ivosidenib and olutasidenib (mutant IDH1-specific inhibitors) and enasidenib (first-in-class mutant IDH2-specific inhibitor). Numerous studies are ongoing to evaluate FDA-approved IDH inhibitors in off-label applications or investigate novel small molecule inhibitors such as the pan-mutant IDH inhibitor vorasidenib [23,24].

So far, gold-standard techniques for *IDH* testing include IDH1 p.R132H-specific immunohistochemistry (IHC), NGS, and PCR-based assays. IHC has the advantage of being easily implemented and automated and can provide results in only a few hours; however, it suffers from subjective interpretations and has shown its inferiority compared to NGS [25]. p.R132H IHC can only detect IDH1 R132H variants, and for this reason, it is only used in routine practice for the characterization of G. NGS methods represent broader approaches that can cover the entire genomic sequence of the *IDH1* and *IDH2* genes and other genes simultaneously, but they are time-consuming and costly and require more expertise than PCR and IHC techniques. Finally, PCR assays represent fast, targeted approaches that can detect several variants at the same time, but they are less exhaustive than NGS and, thus, can miss minor variants.

The Idylla platform (Biocartis, Mechelen, Belgium) is a microfluidic qPCR-based system for the detection of specific molecular alterations with limited hands-on time and no need for nucleic acid extraction beforehand. Idylla KRAS, BRAF, NRAS/BRAF, MSI, and gene fusions cartridges with all reagents on board have been extensively studied and have shown potential for ultrafast testing in specific regions of cancer-associated genes [26,27,28,29,30,31,32,33,34,35]. We and others highlight that extracted DNA can also be pipetted directly into the cartridges to spare supplementary tumor sections, optimize laboratory workflows, and/or retrieve samples whose analysis failed by NGS [36,37,38,39].

**Figure 1:**
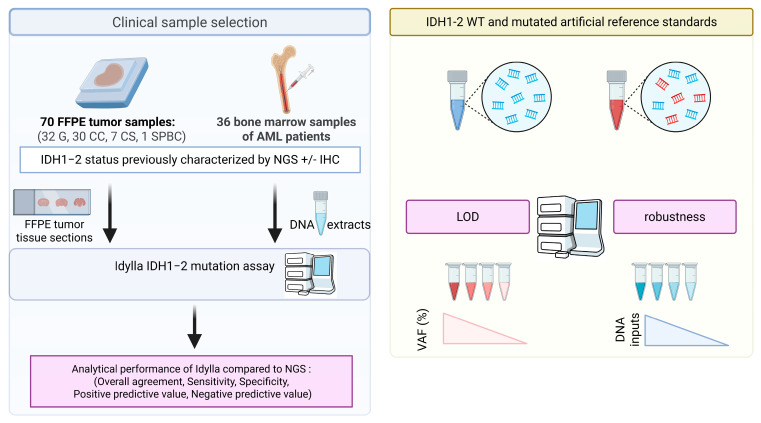
Schematic overview of the study workflow. Abbreviations: AML: acute myeloid leukemia; CC: cholangiocarcinoma; CS: chondrosarcoma; FFPE: formalin-fixed paraffin-embedded; G: glioma; IHC: immunohistochemistry, LOD: limit of detection; NGS: next-generation sequencing, SPBC: solid papillary breast carcinomas with reverse polarity; VAF: variant allelic frequency; WT: wild-type.

In this retrospective study, we evaluated the novel Idylla IDH1-2 mutation assay for the multiplex detection of 5 *IDH1* and 10 *IDH2* gene variants in 106 clinical samples from different tumor types (Figure 1). The results were compared with those previously obtained by targeted NGS during standard routine care. The limit of detection of the Idylla IDH1-2 mutation assay was determined for *IDH1* R132H and *IDH2* R172K variants using a combination of wild-type and mutated commercial reference standards. The robustness of the assay was finally assessed using dilutions of wild-type reference standards to determine the minimal DNA input required for Idylla analysis.

## 2. Results

### 2.1. Analytical Performance of the Idylla IDH1-2 Mutation Assay to Detect Hotspot IDH Variants in Clinical Samples Compared to NGS Reference Techniques

The Idylla IDH1-2 mutation assay provided 104 valid results out of the 106 samples analyzed (98.1%) (Table 1). An uninterpretable result was obtained from the sample CS3. It had been analyzed by Idylla 14 months after sampling and also yielded uninterpretable results by NGS, probably due to low DNA quantity and quality (DNA concentration of 0.8 ng/µL and GQN of 0). The second uninterpretable result was obtained from the sample CC8. CC8 was analyzed by Idylla 11 months after sampling. DNA quantity (2.8 ng/µL) and quality (GQN of 7.0) extracted from this sample were sufficient to provide a valid *IDH* wild-type NGS result. The sample CC8 has not been reanalyzed by Idylla due to tumor tissue depletion (absence of tumor cells on the hematoxylin/eosin-stained post-analytical slide). Therefore, the invalid result obtained from CC8 by Idylla IDH1-2 mutation assay seems to be related to an insufficient amount of tumor material available for the analysis.

Among the 104 cases with valid results, 27 had cycle of quantification (Cq) values for the control targets over 35 (Table 1). In our dataset, no clear association was observed between sample age and control Cq values since older samples did not consistently exhibit higher control Cq values (Supplementary Figure S1). When directly loading tissue sections into the cartridge for Idylla analysis, 39.7% (27/68) of cases harbored suboptimal amplification of the control targets (control Cq values over 35), whereas the use of DNA extracts from AML patients at the input recommended consistently resulted in optimal amplification (control Cq values under 35).

A total of 101 out of the 104 valid results (97.1%) were concordant between the Idylla and NGS reference methods. The discrepant results concerned one glioma (G21), one cholangiocarcinoma (CC6), and one AML (AML1) (Table 2). The G21 sample was found to be *IDH* non-mutated by Idylla, while an *IDH2* R172K variant was detected by NGS with a variant allelic frequency (VAF) of 21%. The initial Idylla analysis showed a control Cq above 35 (38.7), suggesting that the variant may have been missed due to low DNA input or poor quality. A second Idylla analysis was performed using the maximum number of FFPE tissue sections recommended by the manufacturer (four sections), but it yielded the same result (control Cq 37.6). These discordant results may thus be attributed to technical factors, including low DNA concentration (1.4 ng/µL), poor DNA quality (GQN 1.3), and prolonged storage of the sample prior to Idylla testing. In addition, biological factors may also contribute, such as tumor heterogeneity between the different tissue sections used for NGS and Idylla analyses, which could lead to a biological false-negative result even when the variant is present at moderate VAF in the tumor. 

Concerning the AML1 sample, Idylla IDH1-2 mutation assay gave an *IDH* wild-type result (with a control Cq of 31.1) while NGS detected an *IDH1* R132H mutation with VAF of 2%. A second Idylla analysis of the AML1 sample was performed using almost 2-fold higher DNA input (use of 50 µL solution at 22.8ng/µL, i.e., 1,140 ng DNA input) but again provided an *IDH* wild-type status (with a control Cq of 30.7). This discordant result may be due to technical factors, such as the prolonged storage of the sample prior to Idylla analysis (28 months) or the inherent sensitivity limits of the assay. Biological false-negative results cannot be entirely excluded, particularly in this case where the IDH1 variant was detected by NGS at a very low variant allele frequency (VAF).

The CC6 sample was found to be *IDH* non-mutated by NGS, while a variant was detected in codon 172 in the *IDH2* gene (with a mutant Cq of 37.5, a control Cq of 32.3, and a ∆Cq of 5.3). A second Idylla analysis was performed using the same number of tumor sections, and no *IDH* mutation was detected (control Cq of 32.1). Bam files obtained from the NGS analysis were reanalyzed in order to check if a variant could have been missed or filtered, but no supplementary variant in the IDH2 gene was found. The initial Idylla analysis, therefore, appears to have yielded a false-positive result. This highlights the importance of understanding the limitations of the techniques used and, in cases of uncertain results, confirming findings with an orthogonal method, given the potential clinical implications for targeted therapy decisions.

When considering all valid results (*n* = 104) for concordance analysis using NGS as the gold standard, the overall agreement, sensitivity, and specificity of the Idylla IDH1-2 mutation assay were 97.1%, 96.2%, and 98.1%, respectively (Table 3a). The positive and negative predictive values of the Idylla IDH1-2 mutation assay were 98.0% and 96.2%, respectively. A subgroup analysis was also performed to evaluate the specific analytical performance of the Idylla IDH1-2 mutation assay depending on the tumor types. The overall agreement of the Idylla assay was 96.9% for Gs, 96.6% for CCs, 100% for CSs, and 97.2% for AMLs (Table 3b–e). The sensitivity of the assay was 95.8% for Gs, 100% for CCs, 100% for CSs, and 95% for AMLs. The specificity of the IDH1-2 mutation assay was 100% for Gs, 95.8% for CCs, 100% for CSs, and 100% for AMLs. The positive predictive value of Idylla was 100% for Gs, 83.3% for CCs, 100% for CSs, and 100% for AMLs. Finally, the Idylla IDH1-2 mutation assay gave a negative predictive value of 88.9% for Gs, 100% for CCs, 100% for CSs, and 94.1% for AMLs.

### 2.2. Determination of the Limit of Detection (LOD) of the Idylla IDH1-2 Mutation Assay Using Commercial Reference Standards

We determined the LOD of the Idylla IDH1-2 mutation assay using artificial reference standards from Horizon Discovery Ltd (Cambridge, UK). We realized different combinations of *IDH1* and *IDH2* wild-type and mutated standards to obtain a total DNA input of 500 ng and a final volume of 50 µL as recommended by the manufacturer. The LOD of the IDH1-2 mutation assay was defined as 1.6% for the *IDH1* R132H variant and 0.5% for the detection of the *IDH2* R172K (Table 4).

### 2.3. Evaluation of the Robustness of the Idylla IDH1-2 Mutation Assay Using Commercial Reference Standards

**Figure 2:**
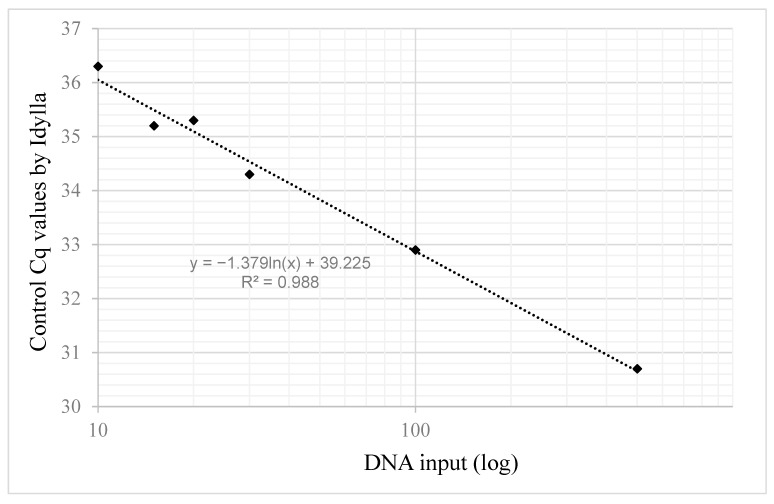
Idylla control Cq values depending on DNA input.

Using reference standards at 10ng total DNA input, the Idylla system succeeded in determining the *IDH* status (Table 5). However, the control Cq values were found to be over 35 for all cases, indicating a suboptimal amount of DNA for amplification. To determine the minimal input of DNA standards for optimal amplification by Idylla, we examined the control Cq obtained with different inputs of the *IDH1* wild-type reference standard (Figure 2). Control Cq was found under 35 starting from 30 ng DNA, indicating that inputs lower than the 500 ng recommended by the provider may be used to detect *IDH* variants by Idylla.

## 3. Discussion

In our study, the Idylla IDH1-2 mutation assay demonstrated high overall concordance with NGS (>95%) for the detection of *IDH* hotspot variants starting from FFPE tumor sections or DNA extracts. Thus, it could represent a valuable alternative to NGS in various tumor types, including gliomas, cholangiocarcinomas, and AML. Our experience on one SPBC sample and seven CS samples also suggests that Idylla IDH1-2 mutation assay could be used in these histological subtypes, albeit deeper multicentric studies are needed to confirm these results.

Nevertheless, several factors should be considered when interpreting Idylla results. Based on the analysis of discordant cases, the most probable causes of false Idylla results were associated with prolonged pre-analytical storage of samples prior to analysis, intrinsic limitations of the Idylla assay (particularly its limit of detection), biological-related factors (including insufficient DNA quantity or quality, or false negative results due to low VAF). Moreover, when loading the recommended number of FFPE tumor tissues sections directly into the cartridges, almost 40% of cases exhibited suboptimal amplification of the control sequences (control Cq over 35). This highlights, in these cases, the limited DNA quantity or quality available for the analysis and the potential risk of missing *IDH* variants with low variant allelic frequencies. Conversely, the use of already extracted DNA for the analysis allows for the evaluation of DNA concentration and adjustment of the input to achieve optimal PCR amplification. For solid tumor samples with low tumor tissue surface, prior DNA extraction and dosage could be performed to have optimal conditions for Idylla analysis. In the same way, particular attention is required for the biological interpretation of the Idylla results when suboptimal amplification of the controls is observed (control Cq values indicated with an asterisk in the Idylla report).

Following the recommendations of the supplier regarding the minimal DNA input to use for the analysis, we determined that the LOD of the Idylla IDH1-2 mutation assay was 1.6% for the *IDH1* R132H variant and 0.5% for the detection of the *IDH2* R172K variant. Robustness assessments showed that DNA input amounts below the recommended levels (down to 30 ng) could be used to obtain optimal amplifications of the target and the controls. For practical and methodological reasons, the analytical limit of detection and robustness of the assay were determined using artificial reference materials. In routine clinical practice, particularly when applied to real-world FFPE samples, the assay’s performance is likely to be moderately reduced, mainly due to the variable tumor cellularity of the specimens and the lower quality and increased fragmentation of DNA typically extracted from such samples.

Contrary to NGS, the Idylla system does not require batch samples and provides results in only 95 min, making it a suitable option for urgent cases. Moreover, it requires less specialized biological expertise for data interpretation and can be more easily implemented in biopathological laboratories compared to NGS approaches. Unlike p.R132H-specific IHC, the Idylla IDH1-2 mutation assay covers multiple IDH variants, although it is less extensive than NGS. Other multiplex PCR-based techniques are also available in the market to detect *IDH* hotspot variants, including IDIDH1-2 (ID Solutions) or the therascreen IDH1/2 RGQ PCR Kit CE (Qiagen). Among all the available PCR approaches, the Idylla IDH1/2 mutation assay has the advantage of being fully automated and requiring minimal hands-on time (<5 min vs. a few hours for most other PCR techniques).

The Idylla IDH1-2 mutation assay has been designed for initial molecular tumor diagnosis and, therefore, cannot detect most *IDH* resistance variants that can emerge during IDH-targeted therapy and cause disease progression—such as *IDH1* S280F and D279N or *IDH2* Q316E and I319M variants—which affect the binding of IDH-mutant inhibitors to their targets [20,40]. Moreover, the Idylla system consists of a targeted PCR assay that is not able to detect variants in receptor tyrosine kinase (RTK) pathway genes (NRAS, KRAS, PTPN11, KIT, and FLT3) associated with innate or acquired resistance to IDH inhibitors [20]. The use of the Idylla IDH1-2 mutation assay during disease monitoring also runs the risk of missing out certain mechanisms of isoform switching from mutant IDH1 to mutant IDH2, or vice versa, which were previously identified as secondary resistance mechanisms [41].

**Figure 3:**
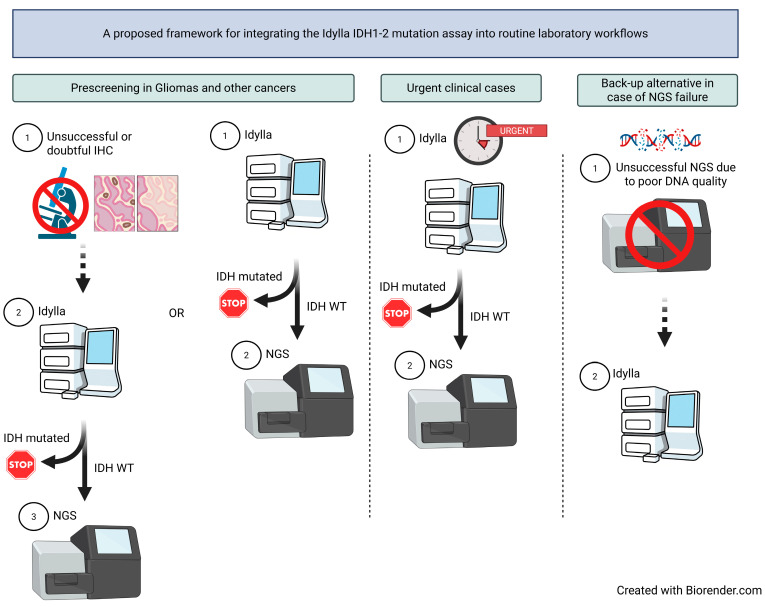
Proposed laboratory workflows integrating the Idylla IDH1-2 mutation assay for initial molecular tumor diagnosis. Abbreviations: IHC: immunohistochemistry, NGS: next-generation sequencing, WT: wild-type.

In the setting of initial diagnosis, the Idylla system may be used as a molecular prescreening tool for gliomas (and other tumor types in which IDH variants and additional relevant alterations are mutually exclusive), either when immunohistochemistry (IHC) yields inconclusive results or as a routine alternative to IHC (Figure 3). The Idylla IDH1/2 mutation assay may also be particularly valuable in clinical situations requiring rapid therapeutic decision-making. In both scenarios, comprehensive NGS should subsequently be performed in cases identified as IDH wild-type by Idylla in order to detect other actionable tumor alterations and facilitate patient access to clinical trials or early drug access programs. The Idylla system may also be advantageous in cases where suboptimal DNA quality precludes the use of broader NGS-based approaches.

Although the prevalence of the different *IDH* variants varies between the tumor types, to our knowledge there is no known difference in terms of the clinical response to IDH-mutant inhibitors depending on the type of variant detected. Thus, the IDH1-2 mutation assay could be sufficient to select patients who will benefit from IDH-targeted therapies.

## 4. Materials and Methods

### 4.1. Tumor Samples Selection

A total of 106 tumor samples were retrospectively selected, including 70 solid tumors and 36 AML. The 70 formalin-fixed paraffin-embedded (FFPE) tumor tissue samples were selected from the biological sample collection of the Institut de Cancérologie de Lorraine (ICL, Nancy, France) and Centre Hospitalier Régional Universitaire de Nancy (CHRUN, Nancy, France). These were obtained from patients with either gliomas (Gs, *n* = 32), cholangiocarcinomas (CCs, *n* = 30), chondrosarcomas (CSs, *n* = 7), or solid papillary breast carcinoma with reverse polarity (SPBC, *n* = 1). Tissue samples were fixed with 10% neutral phosphate-buffered formalin (NBF) for 8–48 h (depending on the size of the tissue specimen), then embedded in paraffin to obtain FFPE blocks. A minimum of 10% tumor cell content was required in whole-slide section or in macrodissected area. The 36 AML specimens consisted of DNA extracted from bone marrow samples of AML patients collected in ethylenediaminetetraacetic acid (EDTA) tubes. Only DNA extracts with a minimal volume of 50 μL and a concentration of at least 10 ng/μL were included. All samples were collected during standard clinical care for patient cancer management. The clinical and biological characteristics of the patients and their tumors are detailed in Table 6. This study was approved by the internal scientific committees of the Institut de Cancérologie de Lorraine and Centre Hospitalier Régional Universitaire de Nancy (N° 2023-05471 and 23/250, respectively). This study was conducted in accordance with the Declaration of Helsinki. All patients included in this study were informed and not opposed to the use of their biological material for research purposes. The need for written informed consent to participate and ethical review and approval was deemed unnecessary according to national regulations (https://www.legifrance.gouv.fr/codes/article_lc/LEGIARTI000041721161, accessed on 18 September 2024). The dates when the data were accessed for research purposes were from 19 June 2023 to 26 February 2024. Data were anonymized at the time of inclusion.

### 4.2. Custom Capture-Based NGS (51-Gene Panel) for Solid Tumor Samples

DNA was extracted from FFPE samples using the QIAamp Generead DNA FFPE kit or QIAamp DNA FFPE advanced UNG kit (QIAGEN, Les Ulis, France). DNA concentrations were determined using the Qubit dsDNA HS assay kit and the Qubit 3.0 Fluorometer (ThermoFisher Scientific Inc., Courtaboeuf, France), following the manufacturer’s instructions. DNA quality was assessed using the Fragment analyzer system (Agilent, Santa Clara, CA, USA), and a genomic quality number (GQN) value ranging from 0 (highly degraded DNA) to 10 (high-quality DNA) was determined for each sample. Targeted NGS analyses were performed using 100 ng of DNA extracted from FFPE samples and a custom capture-based “Solid Tumor Solution” kit (SOPHiA GENETICS, Saint-Sulpice, Switzerland). This 51-gene panel covers regions of potential theranostic interest in the AKT1, ALK, ARID1A, BRAF, BRCA 1, BRCA 2, CDK4, CDKN2A, CTNNB1, DDR2, DICER1, EGFR, ERBB2, ERBB4, ESR1, FBXW7, FGFR1, FGFR2, FGFR3, FOXL2, GNA11, GNAQ, GNAS, H3F3A, H3F3B, HIST1H3B, HRAS, IDH1, IDH2, KIT, KMT2A, KMT2D, KRAS, MAP2K1, MAP2K2, MET, MTOR, MYOD1, NRAS, PDGFRA, PIK3CA, PTPN11, RAC1, RAF1, RET, ROS1, SF3B1, SMAD4, TERT, TGFBR2, and TP53 genes, as previously described [28]. A MiSeq instrument (Illumina, San Diego, CA, USA) was used for sequencing, and the NGS raw data were analyzed using SOPHiA DDM software (pipeline v5.5.89, SOPHiA GENETICS). A minimum of 300× read depth and 95% coverage were required for each sample analyzed.

### 4.3. Commercial Capture-Based NGS (32-Gene Panel) for AML Samples

DNA was previously extracted from blood of patients with AML using the QIAamp DNA blood mini-Kit and QIAcube (QIAGEN). DNA concentrations were determined using the Qubit dsDNA HS assay kit and the Qubit 2.0 Fluorometer (ThermoFisher Scientific Inc., Courtaboeuf, France) or Infinite Pro F200 multimodal plate reader (TECAN, Männedorf, Switzerland), following the manufacturer’s instructions. Targeted NGS analysis was performed using 200 ng of DNA input and the Myeloid Solution MYS kit (SOPHiA GENETICS, Saint Sulpice, Switzerland) following the manufacturer’s recommendations. This panel covers 30 genes of interest, including regions of interest on ABL1, ASXL1, BRAF, CALR, CBL, FLT3, HRAS, IDH1, IDH2, KIT, KRAS, MPL, NPM1, NRAS, PTPN11, SETBP1, SF3B1, SRSF2, U2AF1, WT1, and ZRSR2 genes, and all exons of the CEBPA, CSF3R, DNMT3A, ETV6, EZH2, JAK2, RUNX1, TET2, and TP53 genes [42]. Sequencing was performed on NextSeq 550 instruments (Illumina, San Diego, CA, USA) in a 2 × 150 bp read length and with v2 or v2.5 reagent kits. Analysis of the NGS data was performed using SOPHiA DDM software (SOPHiA GENETICS). A minimal read depth of 1000× and 99% coverage was required for each sample. A genomic reference DNA (Myeloid DNA Reference Standard, Horizon Discovery Ltd., Cambridge, UK) is used to validate each step from library preparation to variant calling.

### 4.4. Biological Characteristics of the Selected Samples

For all samples, the analysis of IDH1 and IDH2 genes was previously performed by NGS during routine clinical care. The biological characteristics of the samples are detailed in Table 1. A total of 32 out of the 70 FFPE samples (45.7%) analyzed harbored an IDH1 or IDH2 variant according to the NGS assay, including 24 Gs (75% of G cases), 5 CCs (16.7%), 2 CSs (28.6%), and 1 SPBC (100%). All G samples found IDH1 R132H mutated by NGS were also p.R132H IHC-positive. Among the 25 G samples non-IDH1 R132H mutated by NGS, 3 were found positive by IHC, with the percentage of stained cells under 5%, while the remaining were IHC-negative. Due to their poor prevalence, only one case of SBPC was included in this study. Among the 36 AML samples analyzed, 16 (44.4%) were found IDH-non-mutated, 8 (22.2%) were IDH1-mutated, and 12 (33.3%) were IDH2-mutated.

#### 4.4.1. Idylla IDH1-2 Mutation Assay

The Idylla platform (Biocartis NV, Mechelen, Belgium) is an automated system that integrates all process steps for real-time PCR assay in single-use cartridges, including sample liquefaction, cell lysis, nucleic acid extraction, PCR amplification, and fluorophore-based detection. The Idylla IDH1-2 mutation assay is a newly developed cartridge for the multiplex PCR-based analysis of IDH1 and IDH2 genes. This assay allows the detection of 5 variants in codon 132 of the IDH1 gene and 10 variants in codons 140 and 172 of the IDH2 gene (see details in Supplementary Table S1) with a turnaround time of approximately 95 min and reduced hands-on time. The entire vial solution, containing primers and unlabeled probes, was loaded into the DNA cartridge (Biocartis). Then, 5-micrometer-thick FFPE sections (for solid tumor samples) or extracted DNA (for hematological cancer samples) were loaded into the cartridge before its launch in the Idylla instrument. If needed, FFPE tumor samples were macrodissected to contain at least 10% tumor content, and a total of 2–4 sections were used, depending on the tissue surface. After the collection of the tumor sections for Idylla analysis, an experienced pathologist validated a final tissue section to determine the tumor content after hematoxylin–eosin coloration. DNA extracted from AML samples was diluted in nuclease-free water to obtain 50 µL of intermediate solution at 12ng/µL for Idylla analysis.

A conserved region in the KIF11 gene is also amplified in each chamber of the cartridge and serves as an internal control. In case of control Cq over 35, the Idylla system indicates that variants with low allelic frequency may not be detected due to low DNA input or quality. If the controls were not amplified, the assay yields an “invalid” result. If a variant is detected, the report indicates the mutant Cq value and ∆Cq (defined as the difference between mutant Cq and control Cq values).

#### 4.4.2. Immunohistochemistry (IHC)

IDH1 p.R132H IHC is used for routine histopathological characterization of Gs following the recommendations of the 2016 WHO classification of tumors of the central nervous system [43,44]. Briefly, 5-micrometer-thick unstained FFPE tumor sections were subjected to deparaffinization and rehydration, then antigen retrieval was obtained using EnVision FLEX Target Retrieval Solution, low pH (97 °C, 40 min), and an EnVision FLEX system (Agilent)***.*** Staining was performed using the anti-IDH1 p.R132H antibody (H09 clone, dilution 1:35, 40 min incubation, Dianova, Hamburg, Germany) and the Dako Omnis instrument (Agilent). Endogenous peroxidases were blocked with EnVision FLEX Peroxidase-Blocking Reagent (3 min incubation, Agilent). Envision Flex + Mouse linker (10 min incubation, Agilent) was used as secondary antibody, then slides were incubated with Envision FLEX linked with HRP (20 min incubation, Agilent). Finally, the staining was visualized using Envision Flex Substrate Working Solution (5 min incubation, Agilent). Slides were counterstained with hematoxylin. Positive and negative internal or external controls were used to check the staining patterns. IHC analysis was performed by a senior pathologist, and results expressed as a percentage of stained cells. Representative IHC images of IDH1 p.R132H-negative and -positive cases are presented in Supplementary Figure S2.

#### 4.4.3. Limit of Detection (LOD) and Robustness of the Idylla IDH1-2 Mutation Assay

Different commercial DNA standards from Horizon Discovery Ltd. were used to determine the limit of detection and the robustness of the Idylla IDH1-2 mutation assay. They include the IDH2 wild-type Reference Standard (reference HD678), IDH2 wild-type Reference Standard (reference HD681), and IDH1 R132H (reference HD677) and IDH2 R172K Reference Standards (reference HD680) with predefined allelic frequencies of 50%. All standards are composed of genomic DNA derived from human cell lines at a concentration of 50 ng/µL in Tris–EDTA (10 mM Tris–HCl, 1 mM EDTA, pH 8.39). To determine LOD, 50 µL solutions were prepared using different volumes of standards and DNAse-free water and loaded into the cartridges prior to the launch of the analyses. All the solutions were prepared to obtain a total DNA input of 500 ng, as recommended by the manufacturer. The LOD was determined as the lowest input yielding a positive result. The robustness of the assay was evaluated using the reference standards diluted in DNAse-free water to obtain DNA inputs lower than those recommended by the supplier (<500 ng).

### 4.5. Statistical Analysis

The sensitivity (Se), specificity (Sp), positive predictive value (PPV), negative predictive value (NPV), and overall agreement (OA) of the Idylla IDH1-2 mutation assay were determined based on the results of IDH status in 106 clinical samples from various tumor types, using NGS standard routine analyses as the gold standard. A 95% confidence interval (95% CI) was determined for all of these parameters using Wilson’s method [45,46].

## Figures and Tables

**Table 1:** IDH status of the 106 clinical specimens obtained by immunohistochemistry (IHC), next-generation sequencing (NGS), and Idylla IDH1-2 mutation assays. Abbreviations: BM: bone marrow; BP: biopsy; Cq: cycle of quantification; NA: not available; NGS: Next-Generation Sequencing; R132H−: absence of p.R132H-mutated protein by IHC; R132H+: detection of p.R132H-mutated protein by IHC; SR: surgical resection; VAF: variant allelic frequency; WT: wild-type.

ID Sample	Sample Age (Months)	Type of Sample	Cell Content [%]	NGS				IDH1 p.R132H IHC		Idylla			
				DNA Concentration (ng/µL)	DNA Quality	IDH Status	VAF	p.R132H-Mutated Protein	% Positive Cells	IDH Status	Mutant Cq	Control Cq	
					(GQN Values)								
GLIOMAS													
G1	6	SR	50%	8.7	4.8	IDH1 R132H	42%	R132H+	100%	IDH1 R132C/H/G/S/L	37.8	33.3	
G2	26		80%	16.5	2.5		41%	R132H+	70%		37.0	32.3	
G3	23		70%	19.9	7.4		43%	R132H+	90%		36.3	32.2	
G4	29		40%	14.9	8.3		41%	R132H+	100%		37.1	33.2	
G5	33		25%	33.4	7.1		14%	R132H+	75%		40.2	33.8	
G6	33		70%	45.5	7.5		32%	R132H+	80%		36.8	32.1	
G7	58		50%	87.4	8.3		37%	R132H+	50%		36.6	31.9	
G8	9		80%	44.4	8.9	IDH1 R132C	39%	R132H−	-		35.8	31.4	
G9	13		60%	23.9	9.1		29%	R132H−	-		38.6	34.3	
G10	12		50%	7.4	7.1		37%	R132H−	-		40.8	36.5 *	
G11	28		60%	54.4	6.6		35%	R132H−	-		35.9	31.6	
G12	40		80%	12.8	0.6		40%	R132H+	1%		37.3	31.7	
G13	44		60%	44.6	2.1		30%	R132H−	-		39.2	36.3 *	
G14	40	BP	60%	4.6	0.3	IDH1 R132L	28%	R132H+	<5%		38.5	36.4 *	
G15	10	SR	40%	71.8	8.1		43%	R132H−	-		34.2	30.5	
G16	53	BP	70%	1.3	4.4	IDH1 R132G	33%	R132H−	-		38.9	38.6 *	
G17	52	SR	60%	7.9	2.3		60%	R132H−	-		36.1	33.5	
G18	98		80%	34.7	4.7		39%	R132H−	-		35.9	33.1	
G19	1		60%	78.9	9.3	IDH1 R132S	43%	R132H−	-		32.3	32.3	
G20	129		75%	7.2	1.6	IDH2 R172K	40%	R132H−	-	IDH2 R172K/M/G/S/W	34.2	37.2 *	
G21	205		50%	1.4	1.3		21%	R132H−	-	WT	-	38.7 *	
G22	101		90%	12.1	0.3	IDH2 R172M	45%	R132H−	-	IDH2 R172K/M/G/S/W	31.8	32.8	
G23	40		80%	48.2	0.0		37%	R132H+	2%		33.0	32.4	
G24	27		80%	38.5	8.2	IDH2 R172G	34%	R132H−	-		35.9	33.4	
G25	30	BP	30%	5.2	8.4	WT	-	R132H−	-	WT	-	36.8 *	
G26	35		80%	15.3	NA		-	R132H−	-		-	38.5 *	
G27	41	SR	30%	3.8	1.1		-	R132H−	-		-	36.3 *	
G28	43	BP	90%	16.5	0.7		-	R132H−	-		-	34.5	
G29	44		30%	2.6	1.0		-	R132H−	-		-	35.1 *	
G30	45		20%	7.7	4.0		-	R132H−	-		-	38.1 *	
G31	48		70%	18.4	1.3		-	R132H−	-		-	36.3 *	
G32	49		30%	3.1	1.2		-	R132H−	-		-	37.6 *	
CHOLANGIOCARCINOMAS													
CC1	10	BP	30%	12.9	6.5	IDH1 R132C	32%	-	-	IDH1 R132C/H/G/S/L	37.9	33.4	
CC2	78	SR	40%	103.0	0.1		18%	-	-		35.4	29.8	
CC3	38	BP	80%	7.2	NA	IDH1 R132S	24%	-	-		37.8	36 *	
CC4	51	SR	30%	39.1	8.3	IDH2 R172K	26%	-	-	IDH2 R172K/M/G/S/W	36.0	33.5	
CC5	58	BP	80%	6.8	0.5		39%	-	-		34.5	36.6 *	
CC6	51	SR	30%	84.1	6.4	WT	-	-	-		37.5	32.3	
CC7	9	BP	80%	4.8	7.3		-	-	-	WT	-	34.6	
CC8	11		50%	2.8	7.0		-	-	-	Invalid	-	-	
CC9	42	SR	30%	42.1	8.1		-	-	-	WT	-	33.9	
CC10	15		15%	22.7	9.1		-	-	-		-	31.1	
CC11	13	BP	50%	0.9	6.3		-	-	-		-	37.8 *	
CC12	74	SR	30%	33.4	3.1		-	-	-		-	30.5	
CC13	9	BP	20%	1.8	8.3		-	-	-		-	34.1	
CC14	58	SR	85%	48.3	2.0		-	-	-		-	30.3	
CC15	19	BP	70%	3.9	8.0		-	-	-		-	35.8 *	
CC16	18		20%	5.6	7.2		-	-	-		-	34.2	
CC17	20	SR	70%	17.4	3.0		-	-	-		-	31.0	
CC18	18	BP	50%	10.9	8.3		-	-	-		-	35.4 *	
CC19	17		70%	5.7	7.4		-	-	-		-	35.2 *	
CC20	20	SR	70%	98.6	3.8		-	-	-		-	30.2	
CC21	3	BP	60%	53.2	9.5		-	-	-		-	36.3 *	
CC22	11	SR	20%	64.8	7.1		-	-	-		-	29.8	
CC23	2	BP	60%	62.1	9.6		-	-	-		-	33.3	
CC24	3		70%	12.5	9.6		-	-	-		-	35.1 *	
CC25	1		70%	7.8	9.5		-	-	-		-	33.0	
CC26	4	SR	30%	143.0	8.7		-	-	-		-	31.0	
CC27	19		40%	161.5	6.8		-	-	-		-	29.2	
CC28	1	BP	10%	16.7	9.5		-	-	-		-	35.3 *	
CC29	2	SR	30%	40.5	8.5		-	-	-		-	34.1	
CC30	1	BP	40%	5.9	9.4		-	-	-		-	35.0	
CHONDROSARCOMAS													
CS1	5	BP	60%	2.1	9.5	IDH1 R132L	6%	-	-	IDH1 R132C/H/G/S/L	42.4	35.1 *	
CS2	7	SR	60%	8.6	8.7	IDH1 R132C	8%	-	-		39.9	34.6	
CS3	14	SR	90%	0.8	0.0	Invalid	-	-	-	Invalid	-	-	
CS4	3	BP	25%	5.8	9.6	WT	-	-	-	WT	-	36.2 *	
CS5	15	BP	60%	0.8	7.9		-	-	-		-	38.2 *	
CS6	0	BP	30%	4.2	9.6		-	-	-		-	38.2 *	
CS7	0	BP	80%	5.0	9.7		-	-	-		-	38.3 *	
SOLID PAPILLARY BREAST CARCINOMA WITH REVERSE POLARITY													
SPBC1	3	SR	80%	182.0	8.8	IDH2 R172S	33%	-	-	IDH2 R172K/M/G/S/W	33.1	31.0	
ACUTE MYELOID LEUKEMIAS													
AML1	28	BM	13%	76.2	-	IDH1 R132H	2%	-	-	WT	-	31.1	
AML2	17		94%	130.0	-		48%	-	-	IDH1 R132C/H/G/S/L	32.3	28.4	
AML3	9		88%	117.0	-		49%	-	-		32.4	28.7	
AML4	9		20%	154.6	-		45%	-	-		32.7	28.0	
AML5	11		74%	140.2	-	IDH1 R132C	47%	-	-		32.1	28.4	
AML6	23		88%	75.8	-	IDH1 R132G	36%	-	-		30.4	30.3	
AML7	17		40%	27.0	-		29%	-	-		31.8	30.2	
AML8	8		0%	24.5	-		24%	-	-		32.0	30.3	
AML9	7		91%	40.0	-	IDH2 R140Q	49%	-	-	IDH2 R140Q/L/G/W	28.0	30.3	
AML10	8		93%	277.7	-		45%	-	-		32.0	30.1	
AML11	24		83%	144.6	-		51%	-	-		28.3	30.5	
AML12	26		55%	41.7	-		40%	-	-		28.9	30.8	
AML13	16		52%	184.0	-		49%	-	-		27.7	29.8	
AML14	14		63%	130.9	-		44%	-	-		29.8	29.4	
AML15	9		82%	108.7	-		48%	-	-		28.7	30.8	
AML16	12		29%	99.0	-		41%	-	-		32.5	30.3	
AML17	17		14%	62.5	-		21%	-	-		30.0	30.0	
AML18	21		39%	156.9	-	IDH2 R172K	30%	-	-	IDH2 R172K/M/G/S/W	30.7	29.8	
AML19	18		83%	47.6	-		40%	-	-		31.2	31.1	
AML20	10		64%	43.3	-		31%	-	-		31.5	30.4	
AML21	14		71%	37.5	-	WT	-	-	-	WT	-	31.2	
AML22	13		78%	226.0	-		-	-	-		-	29.0	
AML23	12		93%	298.6	-		-	-	-		-	30.1	
AML24	8		92%	260.6	-		-	-	-		-	29.6	
AML25	8		35%	27.8	-		-	-	-		-	30.6	
AML26	8		96%	147.1	-		-	-	-		-	30.3	
AML27	7		10%	163.0	-		-	-	-		-	30.1	
AML28	7		35%	175.2	-		-	-	-		-	29.8	
AML29	7		21%	85.6	-		-	-	-		-	30.5	
AML30	7		16%	61.0	-		-	-	-		-	30.7	
AML31	7		9%	118.0	-		-	-	-		-	29.7	
AML32	6		81%	344.9	-		-	-	-		-	30.2	
AML33	5		92%	68.0	-		-	-	-		-	29.1	
AML34	5		81%	170.0	-		-	-	-		-	29.3	
AML35	5		85%	362.0	-		-	-	-		-	30.3	
AML36	4		13%	157.0	-		-	-	-		-	30.2	

* indicates that the control Cq is over 35. In these cases, the Idylla system warns that variants with low allelic frequency may not be detected due to low DNA input or quality.

**Table 2:** Discrepant results between next-generation sequencing (NGS) +/− immunohistochemistry (IHC) and Idylla IDH1-2 mutation assays. Abbreviations: AML: acute myeloid leukemia; BM: bone marrow; NGS: Next-Generation Sequencing; R132H−: absence of p.R132H-mutated protein by IHC; SR: surgical resection; VAF: variant allelic frequency, WT: wild-type.

ID Sample	Sample Age (Months)	Type of Sample	Tumor Cell/Blast Content [%]	NGS				IDH1 p.R132H IHC	Idylla			
				DNA Concentration (ng/µL)	DNA Quality	IDH Status	VAF	p.R132H-Mutated Protein	IDH Status		Mutant Cq	Control Cq
					(GQN Values)							
G21	205	SR	50%	1.4	1.3	IDH2 R172K	21%	R132H−	1st analysis (3 FFPE sections)	WT	-	38.7 *
									2nd analysis (4 FFPE sections)	WT	-	37.6 *
CC6	51	SR	30%	84.1	6.4	WT	-	-	1st analysis (3 FFPE sections)	IDH2 R172K/M/G/S/W	37.5	32.3
									2nd analysis (3 FFPE sections)	WT	-	32.1
AML1	28	BM	13%	76.2	-	IDH1 R132H	2%	-	1st analysis (600 ng DNA input)	WT	-	31.1

* indicates that the control Cq is over 35. In this case, the Idylla system warns that variants with low allelic frequency may not be detected due to low DNA input or quality.

**Table 3:** Concordance of IDH status between Idylla and NGS results for (**a**) cases from all tumor types, (**b**) gliomas, (**c**) cholangiocarcinomas, (**d**) chondrosarcomas, and (**e**) AMLs. Sensitivity (Se), specificity (Sp), positive predictive value (PPV), negative predictive value (NPV), and overall agreement (OA) were calculated for Idylla using NGS routine analyses as the gold standard. The 95% confidence intervals are provided within square brackets. Abbreviations: +: detection of an IDH variant; −: absence of an IDH variant; NPV: negative predicate value; OA: overall agreement; PPV: positive predicate value; NGS: next-generation sequencing; Se: sensitivity; Sp: specificity, *: gold standard.

(**a**) All cases		Idylla		Total
		+	−	
NGS *	+	50	2	52
	−	1	51	52
Total		51	53	104
OA: 97.1% [91.9; 99.0] Se: 96.2% [87.0; 98.9] Sp: 98.1% [89.9; 99.7]			PPV: 98.0% [89.7; 99.7] NPV: 96.2% [87.3; 99.0]	
(**b**) Gliomas		Idylla		Total
		+	−	
NGS *	+	23	1	24
	−	0	8	8
Total		23	9	32
OA: 96.9% [84.7; 99.5] Se: 95.8% [79.8; 99.3] Sp: 100.0% [67.6; 100.0]			PPV: 100.0% [85.7; 100.0] NPV: 88.9% [56.5; 98.0]	
(**c**) Cholangiocarcinomas		Idylla		Total
		+	−	
NGS *	+	5	0	5
	−	1	23	24
Total		6	23	29
OA: 96.6% [82.8; 99.4] Se: 100.0% [56.6; 100.0] Sp: 95.8% [79.8; 99.3]			PPV: 83.3% [43.7; 97.0] NPV: 100.0% [85.7; 100.0]	
(**d**) Chondrosarcomas		Idylla		Total
		+	−	
NGS *	+	2	0	2
	−	0	4	4
Total		2	4	6
OA: 100.0% [61.0; 100.0] Se: 100.0% [34.2; 100.0] Sp: 100.0% [51.0; 100.0]			PPV: 100.0% [34.2; 100.0] NPV: 100.0% [51.0; 100.0]	
(**e**) AML		Idylla		Total
		+	−	
NGS *	+	19	1	20
	−	0	16	16
Total		19	17	36
OA: 97.2% [85.8; 99.5] Se: 95.0% [76.4; 99.1] Sp: 100.0% [80.6; 100.0]			PPV: 100.0% [83.2; 100.0] NPV: 94.1% [73.0; 99.0]	

**Table 4:** Limit of detection (LOD) of the Idylla IDH1-2 mutation assay for the detection of IDH1 R132H and IDH2 R172K hotspot variants using artificial reference standards.

IDH Variant	Ratio IDH Mutant Copies/Total IDH Copies (%)	Idylla Results			
		Mutant Cq	Control Cq	∆Cq	Biological Interpretation
IDH1 R132H	10%	35.4	30	5.4	Detected
	5%	36.3	30.2	6.4	Detected
	2.5%	37	30.2	6.9	Detected
	1.6%	37.4	30.3	7.5	Detected
	1%	/	30.2	/	Not detected
IDH2 R172K	10%	33	31.1	3.4	Detected
	5%	33.9	30.5	3.6	Detected
	1.6%	35.9	30.5	5.4	Detected
	1%	36.5	30.5	6	Detected
	0.5%	36.8	30.3	6.5	Detected
	0.25%	/	30.5	/	Not detected

**Table 5:** Robustness of the Idylla IDH1-2 mutation assay. Abbreviations: Cq: cycle of quantification; WT: wild-type; ∆Cq: difference between mutant Cq and control Cq values. * indicates that the control Cq is over 35. In these cases, the Idylla system warns that variants with low allelic frequency may not be detected due to low DNA input or quality.

IDH Reference Standard	Total DNA Input (ng)	Idylla Results			
		Mutant Cq	Control Cq	∆Cq	Biological Interpretation
IDH1 R132H	10	38.3	36.2 *	3.8	IDH1 R132 Mutated
IDH1 WT	10	/	36.3 *	/	Not mutated
IDH2 R172K	10	35.2	36.1 *	−0.9	IDH2 R172 Mutated
IDH2 WT	10	/	36.0 *	/	Not mutated
IDH1 WT	10	/	36.3 *	/	Not mutated
	15	/	35.2 *	/	Not mutated
	20	/	35.3 *	/	Not mutated
	30	/	34.3	/	Not mutated
	100	/	32.9	/	Not mutated
	500	/	30.7	/	Not mutated

**Table 6:** Clinico-pathologic and molecular characteristics of the cohort studied.

	Gliomas (Gs, *n* = 32)	Cholangiocarcinomas (CCs, *n* = 30)	Chondrosarcomas (CSs, *n* = 7)	Solid Papillary Breast Carcinoma with Reverse Polarity (SPBC, *n* = 1)	Acute Myeloid Leukemias (AMLs) (*n* = 36)	All (*n* = 106)
Sex:						
- Male (*n*, %)	17 (53%)	15 (50%)	4 (57.1%)	/	16 (44.4%)	52 (49.1%)
- Female (*n*, %)	15 (47%)	15 (50%)	3 (42.9%)	1 (100%)	20 (55.6%)	54 (51%)
Age at diagnosis, years:						
median [interquartile range]	45 [34; 49]	68 [57; 74]	65 [58; 77]	78	69 [61; 75]	65 [51; 72]
Cell tumor or blast content (%):						
median [interquartile range]	60% [48%; 80%]	45% [30%; 70%]	60% [45%; 70%]	80%	68% [27%; 86%]	64% [29%; 83%]
Type of specimen (*n*, %):						
- Biopsy	9 (28.1%)	17 (56.7%)	5 (71.4%)	/	/	31 (29.3%)
- Surgical resection	23 (71.9%)	13 (43.3%)	2 (28.6%)	1 (100%)	/	39 (36.8%)
- Bone marrow	/	/	/	/	36 (100%)	36 (34%)
IDH status according to NGS results (*n*, %):						
- IDH1-mutated	19 (59.4%)	3 (10%)	2 (28.6%)	0 (0%)	8 (22.2%)	32 (30.2%)
- IDH2-mutated	5 (15.6%)	2 (6.7%)	0 (0%)	1 (100%)	12 (33.3%)	20 (18.9%)
- IDH1 and IDH2 wild-type	8 (25%)	25 (83.3%)	4 (57.1%)	0 (0%)	16 (44.4%)	53 (50%)
- Uninterpretable results	0 (0%)	0 (0%)	1 (14.3%)	0 (0%)	0 (0%)	1 (0.9%)

## Data Availability

The dataset presented in the current study is available in a publicly available repository (https://www.ncbi.nlm.nih.gov/bioproject/1177386, accessed on 24 October 2024).

## Supplementary Materials

The following supporting information can be downloaded at https://www.mdpi.com/article/10.3390/ijms27021017/s1.
