# Supplementary material for: Evaluation of the Idylla IDH1-2 Mutation Assay for the Detection of IDH Variants in Solid Tumors and Hematological Malignancies
Source: Int J Mol Sci. 2026 Jan 20;27(2):1017. doi: 10.3390/ijms27021017 (PMC12842437; doi:10.3390/ijms27021017)
Supplement: Supplementary file 1 [file ijms-27-01017-s001.zip › ijms-4052493-supplementary.pdf]

Evaluation of the Idylla IDH 1-2 mutation assay for the detection of *IDH* variants in solid tumors and hematological malignancies

Pauline Gilson, Marc Muller, Guillaume Gauchotte, Smahane Fadil, Marie Husson, Idrissia Hanriot, Andréa Witz, Julie Dardare, Margaux Betz, Jean-Louis Merlin, Alexandre Harlé

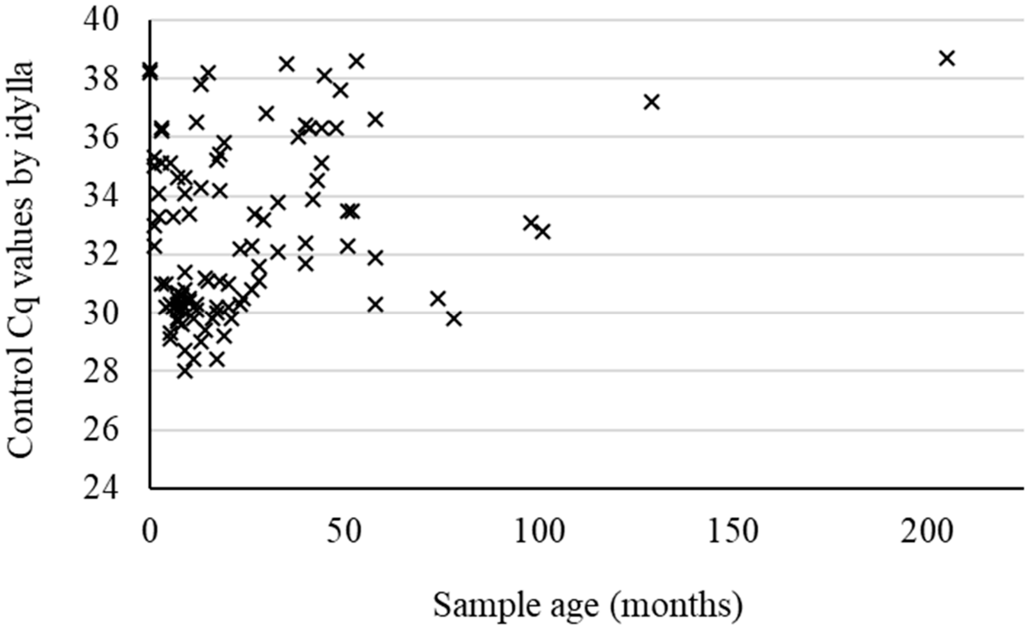

Supplementary Figure S1. Idylla control Cq values depending on sample age.

Supplementary Table S1. ***IDH*** **variants covered by the Idylla IDH 1-2 mutation assay.**

All informations for users concerning the Idylla IDH 1-2 mutation assay can be obtained at the following link: https://www.biocartis.qarad.eifu.online/BCT (BCT019637).

Gene	Codon	Nucleotide change	Protein alteration	Variant
IDH1	132	c.394C>T	p.(Arg132Cys)	R132C
		c.395G>A	p.(Arg132His)	R132H
		c.394C>G	p.(Arg132Gly)	R132G
		c.394C>A	p.(Arg132Ser)	R132S
		c.395G>T	p.(Arg132Leu)	R132L
IDH2	140	c.419G>A	p.(Arg140Gln)	R140Q
		c.419G>T	p.(Arg140Leu)	R140L
		c.418C>G	p.(Arg140Gly)	R140G
		c.418C>T	p.(Arg140Trp)	R140W
	172	c.515G>A	p.(Arg172Lys)	R172K
		c.515G>T	p.(Arg172Met)	R172M
		c.514A>G	p.(Arg172Gly)	R172G
		c.516G>T ; c.516G>C	p.(Arg172Ser)	R172S
		c.514A>T	p.(Arg172Trp)	R172W

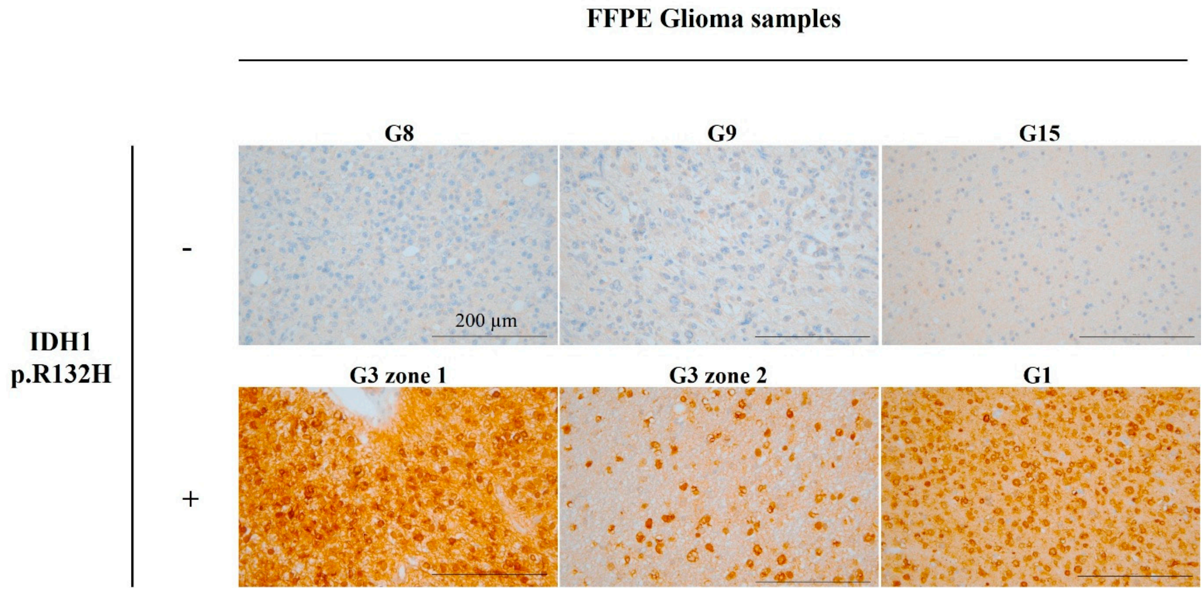

Supplementary Figure S2. Representative IHC images of IDH1 p.R132H–negative and –positive cases

Scale bar = 200µm.

Abbreviations: FFPE: formalin-fixed paraffin-embedded.
